# The Domestic Staff—II

**Published:** 1908-08-29

**Authors:** 


### Nursing Administration.

### THE DOMESTIC STAFF.—II.

### SUPERINTENDENCE.

Having got rid of the scrubber or outside worker and gathered the domestic staff under the wing of the institution, it becomes a matter of supreme importance to bring them under discipline, train them in their work, and ensure that it is always kept up to the mark. This duty must be performed by one person, who is responsible for the way the work is done right through the institution. In small hospitals there is no doubt that this labour must rest on the matron herself, but, then, in small hospitals the staff is a small one and the difficulties of superintendence are proportionately lessened. There is no better training possible for young servants than the personal attention of a busy woman who thoroughly understands how the work ought to be done, and who has learned how to combine domestic inspection with her other multifarious duties. In the eyes of an experienced matron the ignorant, helpless young woman in whose defects less capable people would find much to complain about is so much raw material ready to be moulded, and she no more expects to see her improve without the usual application of counsel and instruction than a potter expects the clay to fall of its own accord into cups and basins ready for use. Some clays are more malleable than others: that is the difference. In a small household it is comparatively easy to set a tone; there is more chance for the maids of getting a variety of work; and provided that the matron regards their training as a duty instead of letting even the single-handed maid muddle along in any ways she happens to have fallen into, the domestic atmosphere ought to be perfectly serene. But there are many hospital households in which the claims on the matron's time prevent her from giving sufficient time to the efficient training of her domestic staff. The number of servants is too great, the size of the institution too large, to enable her to watch the work and inspect it as it ought to be inspected. It is under these conditions, when the matron is at the same time unprovided with the help of an assistant matron, that domestic difficulties assume their most serious dimensions. A tacit and most dangerous understanding is apt to prevail that every servant knows, or ought to know, her own duties, and that the only thing necessary in the interests of those whose comfort depends on their ministrations is to scold. So the sisters scold the wardmaids, the nurses complain of their dormitory maids, the matron finds fault with the head housemaid, and everyone (*sotto voce*) abuses the cook. The best thing to be done under these circumstances is to exalt the position of the cook to that of cook-housekeeper and engage a superior woman who could be responsible for the behaviour and work of the other servants, under the general superintendence of the matron. A superior upper servant who has learned the art of training often manages better than anyone else with the class of girls who offer themselves as institution servants. She is always on the spot, she understands their little ways, and with the broad outlines of the system laid down for her by the matron she is likely to maintain the discipline and harmony of the household. But she must be well chosen, well paid, and well trusted. A few extra pounds added on to the cook's wages would be, indeed, an economy could the result we have indicated be thus easily achieved.

In large institutions the training of the domestic staff falls naturally to the assistant matron, the home sister being responsible, perhaps, for the staff in the nursing home. It is a valuable part of the training afforded in big institutions that it offers initiation into the details of domestic organisation, and sisters who wish to take up matrons' appointments are eager to gain experience in this direction.

But whether it be the matron herself, or some upper servant, or the assistant matron on whom the responsibility falls of superintending the servants, the main system must in each case be the same if order and contentment are to be maintained. Each maid must have a time-table of her work, which must be revised at stated intervals and be strictly adhered to. A well-thought-out time-table for each day of the week, conscientiously carried out from day to day, is the hinge on which the whole comfort of the establishment depends. To draw it out is a work requiring great attention to detail and constant consideration of seasonal alterations. In the hands of a blundering housekeeper the time-table is nothing but a snare and a stumbling-block. All neglected duty is ascribed to it; when anything out of the common needs to be done the time-table will always block the way. The result is that in some places it has fallen altogether into disuse. True, the time-table may easily be at fault, but, then, it is easily rectified; and when constructed on good lines, so as to supply a backbone to the entire institution, it is found indispensable. It is the groundwork of all punctuality. It must be as familiar to the housekeeper as to the maids. By aid of it she inspects the work day by day, not as a matter of fault-finding, but as part of the routine, and will find that as order becomes familiar so it becomes loved by her staff. Young servants are very much the creatures of habit, and when their superior has their confidence it is surprising how soon they learn to take pleasure in the beaten track and in the smooth performance of simple duties. It is one thing to toil many undefined hours cleaning boots, about which nothing will ever be heard unless the echo of some frantic grumble penetrates to the kitchen, and another to keep an eye on the clock in order that an incredibly long array of brightly polished boots may be ready for the inspection of "Sister," and possibly her approving smile, at a given moment. But the superintendent must be prepared for much more than the superintendence of work if she is to find satisfaction in her staff. *(To be continued.)*

